# Sexual Practices and Their Development Pattern Among Jimma University Students

**DOI:** 10.4314/ejhs.v20i3.69445

**Published:** 2010-11

**Authors:** Fentie Ambaw, Andualem Mossie, Teshome Gobena

**Affiliations:** 1 Department of Health Education and Behavioral Sciences, Jimma University; 2 Department of Physiology, Jimma University

**Keywords:** oral sex, anal sex, Jimma

## Abstract

**Background:**

Traditional views of sexual behaviors are frequently changing as the factors influencing them are changing. Therefore, assessing sexual practices that are not part of the tradition would be necessary. The objective of this study was to identify the types of sexual practices, their development pattern and how these development patterns expose students to sexually transmitted infections and unplanned pregnancy.

**Methods:**

A cross-sectional survey was conducted on a sample of 1986 (1612 males, 365 females, and with 9 subjects' gender not indicated) Jimma university students in August 2009 with their age ranging from 17–45 years (median = 20). Quantitative data was collected using a piloted, precoded questionnaire and qualitative data was collected from six focus group discussions. Logistic regression and descriptive statistics were computed and qualitative findings were triangulated with quantitative findings. P-value less than 0.05 was considered significant.

**Results:**

Practice of penile to vaginal intercourse, masturbation, kissing, oral sex, and anal sex were reported by 567 (28.9%), 688 (36.7%), 840 (42.4%), 179 (9.2%) and 83 (4.3%) of the respondents, respectively. Respondents had two years (one year with and one year without condom) of sexual experience before marriage. Sixty percent of those who had sexual experience were exposed to sexually transmitted infections and 46.6% were exposed to both unplanned pregnancy and sexually transmitted infections. Forty seven percent of those who practiced oral sex and 29% of those who practiced anal sex did not consider their acts as sexual intercourse.

**Conclusions:**

University students are high risk groups that need more focused research and concerted health care. The term ‘sexual intercourse’ should be consciously defined for its future use in Ethiopia. Furthermore, Service providers and researchers should address all types of sexual practices.

## Introduction

Sexual behavior among the adolescent and the youth is the core of sexuality matters for the fact that it affects adult life negatively and, therefore, stimulated many research interests for the need to provide evidence for the discrepancy between the beginning of sexual life and that of conjugal life (1).

Reports are emerging from all over the world about sexual debut age, marriage age, number of sexual partners, and contraceptive and condom use patterns. The Ethiopian Demographic and Health Survey report these indicators every 5 years from a nationally representative data (2).

There are evidences that sexually risk-taking behaviors are influenced by many diverse factors which include poverty, race, ethnicity, and religiosity, puberty age, peer relations, school performance, and curiosity for sex, coercion, family composition and relationships (3). A number of studies also revealed negative consequences of early sexual activity which can have physical, psychological, social, and economic dimensions (4–13).

However, questions concerning the type of sexual practices being practiced by the youth, the existence of pattern in the development of these practices, and the safety of sexual behavior development pattern preventing the subjects from sexually transmitted infections and unplanned pregnancy, were not answered by the available literature satisfactorily. This is specially, the problem in Ethiopia where sexual practice is understood as a heterosexual penile-to-vaginal intercourse. The provision of answers in these aspects would initiate prevention efforts that meet the needs of adolescents and youth with different sexual expectations and experiences (10). It is also important to question out of the traditional views as sexual behaviors are frequently changing with changes in the factors influencing them (14).

The purpose of this study was to identify the type of sexual practices, their development pattern and how the development pattern exposes the subjects to sexually transmitted infections and unplanned pregnancy among Jimma university students.

## Subjects and Methods

The study was conducted in Jimma University-main campus in May 2009. Jimma University is an Institution of Higher Education located in Jimma, 357km southwest of Addis Ababa-Ethiopia. A cross-sectional survey was conducted on a sample of 1986 regular students (from freshman to graduating classes) using both qualitative and quantitative methods of data collection.

Sample size for the quantitative part of the study was calculated using the estimated prevalence of sexual intercourse for the source population which was 32% from a recent study (15). A single population proportion formula was used at a confidence level of 95% and margin error of 3% (16). Since we clustered the source population using lecture classes (average of 65 students), we used a design effect of 2 as recommended by Adamchalk S et al. (17) for populations that are not too divergent from cluster to cluster, resulting in 1858. With 10% contingency, the total sample size for the study was 2044. Then students of 31 lecture classes were randomly selected using lottery method and studied out of a list of 206 clusters. The qualitative part of the study included six focus group discussions with 7–10 purposely selected participants in each group. The groups included both genders and different educational levels. Participants who were included in the quantitative part of the study were not considered for the qualitative part.

The dependent variables were types of sexual practices (operational definition given in brackets): Sexual intercourse (penis to vaginal sex), anal sex (penis to anus sex), oral sex (penis to mouth or mouth to vagina stimulation), masturbation (manipulating one's own genital organ for sexual pleasure), kissing (kissing for sexual pleasure), exposure to STI (ever practice of sexual intercourse without condom), and exposure to unplanned pregnancy (ever practice of sexual intercourse without both condom and contraceptives). The independent variables included age, gender, religion, church/ mosque attending habit, ethnicity, place of origin, current place of residence, years of university study, faculty, and marital status. The variable examined qualitatively was the ‘definition of sexual intercourse.’

The quantitative data was collected by trained Jimma University instructors using a structured, piloted, and precoded questionnaire prepared in English and administered in classrooms arranged for the purpose. The questionnaires were collected as soon as they were completed by the respondents. The qualitative data was collected by two facilitators in each focus group who used tape recorders and took notes to document the discussion. Each focus group lasted 45– 75 minutes.

The quantitative data was analyzed using SPSS for windows version 13.0. Logistic regression and descriptive statistics were computed using p-value of less than 0.05 as statistically significant. Further more, the data from the qualitative part of the study was triangulated with the quantitative findings.

The proposal was reviewed and approved by College of Public Health and Medical Sciences, Jimma University Ethical Review Committee. Informed consent was obtained from each of the participants before data collection.

## Results

A total of 1986 (1612 males, 365 females and with 9 subjects' gender not indicated) students provided valid responses making the response rate for the study 97.2%. The respondents age ranged from 17– 45 years (median= 20 years) with 1444 (74.1%) of them between 20–24 years, 1731 (88%) of them being single, and 1082 (54.6%) of them first year students (table 1).

**Table 1:** Some socio-demographic characteristics of the respondents, Jimma University, August 2009.

Characteristics		Number (per cent)
**Age in years** (n=1949) (mean=20.85±2.2, median=20, Minimum=17, maximum=45		
	17– 19	421 (21.6%)
	20– 24	1444 (74.1%)
	25– 45	84 (4.3%)
**Sex** (n= 1977)	Male	1612 (81.5%)
	Female	365 (18.5%)
**Religion** (n= 1959)		
	Orthodox	1046 (53.4%)
	Muslim	348 (17.8%)
	Protestant	480 (24.5%)
	Others^a^	85 (4.3%)
**Church / mosque attending habit** (n= 1962)		
	Yes	1720 (87.7%)
	No	242 (12.3%)
**Ethnicity** (n= 1945)		
	Amhara	619 (31.8%)
	Oromo	832 (42.8%)
	Tigre	58 (3%)
	Gurage	137 (7%)
	Wolita	52 (2.7%)
	Others^b^	247 (12.7%)
**Place of origin** (n= 1961)		
	Urban	945 (48.2%)
	Rural	1016 (51.8%)
**Current place of residence** (n= 1978)		
	In the university	1858 (93.9%)
	Out of the university	120 (6.1%)
**Level of education** (n= 1982)		
	Year-one	1082 (54.6%)
	Year-two	313 (15.8%)
	Year-three	300 (15.1 %)
	Year-four or above	287 (14.5%)
**Faculty** (n= 1985)		
	Medical Sciences	594 (29.9%)
	Public Health	429 (21.6%)
	Business and Economics	149 (7.5%)
	Technology	97 (4.9%)
	Humanities and Social Sciences	423 (21.3%)
	Law	184 (9.3%)
	Education	109 (5.5%)
**Marital status** (n= 1968)		
	Single	1731 (88%)
	Married	35 (1.8%)
	Have boy/girl friend	202 (10.2%)

Othres^a^ include catholic, “waqofetta”, no religion

Others^b^ include Sidama, Keffa, Silte, Agew, Gamo, Somale, Shinasha, Yem, Dawro, Hamer, hadya

Practice of masturbation, kissing, oral sex, and anal sex were reported by 688 (36.7% (male-42.2% and female-10.7%)), 840 (42.4% (males-42.2% and females-41.6%)), 179 (9.2 % (male-9.8% and female=6.3%)), and 83 (4.3% (male-4.6% and female-2.9%)) of the respondents, respectively. Five hundred sixty seven (28.9%) of the respondents practiced sexual intercourse, among which 338 (59.6%) practiced without condom and 234 (46.6%) without both condom and any other contraceptives (table 2).

**Table 2:** Ever practice of some sexual behaviors of the respondents, Jimma University, August, 2009.

Characteristics		Number (percent)
**Sexual intercourse** (n= 1965)		
	Yes	567 (28.9%)
	No	1141 (57.6%)
**Practice of kissing** (n= 1981)		
	Yes	840 (42.4%)
	No	1141 (57.6%)
**Practice of oral sex** (n= 1945)		
	Yes	179 (9.2%)
	No	1766 (90.8%)
**Practice of anal sex** (n= 1921)		
	Yes	83 (4.3%)
	No	1938 (95.7%)
**Practice of masturbation** (n= 1874)		
	Yes	688 (36.7%)
	No	1186 (63.3%)
**Ever practice of sex without condom** (n= 567)		
	Yes	338 (59.6%)
	No	229 (40.4%)
**Ever practice of sex without both condom and other** **contraceptive** (n=502)	Yes	234 (46.6%)
	No	268 (53.4%)

Eighty four (47.2%) of those who practiced oral sex and 24 (28.9%) of those who practiced anal sex did not consider their acts as sexual intercourse. In line with this quantitative finding, most of the participants in the focus group discussion defined sexual intercourse in the context of the university as ‘a heterosexual penile-vaginal sexual practice’ although they believed the presence of anal sex, oral sex, phone sex, homosexuality and lesbianism. Even some of the participants disclosed their strong disapproval of all other sexual practices except penis to vagina sex. A second year male student described his experience as follow:

What I call sexual intercourse is heterosexual and vaginal. I do not want to hear about anal sex but we have heard that some students are practicing it. Mostly, it is homosexual. A male student asked a friend of mine (male) for sex and even gave him a quick kiss. I was shocked when I heard this event from my friend.

Another first year female student added the following:

*What we call sexual intercourse is heterosexual and vaginal. All other practices are not considered to be sexual intercourse although the ‘ferenges’* (*Whites) call them that way.*

**Figure 1:**
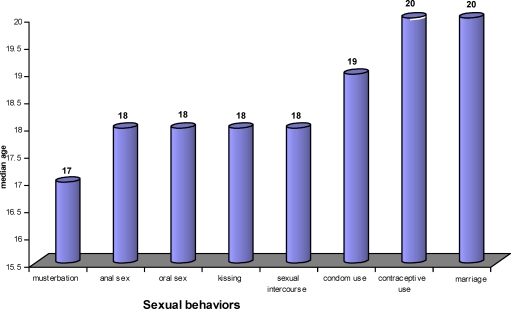
Sexual behavior of the respondents by their median age of initiation, August 2009.

Regarding the development of sexual behaviors, the median age for masturbation was 17 years, the median age for sexual intercourse was18 years (also the same for oral sex and anal sex), the median age for condom use was 19 years, and the median age for marriage was 20 years (same also for contraceptive use) (figure 1).

Logistic regression analysis showed that students in the age group 20–24 and 25–45 years were 13.49; 95%CI, 7.63–23 and 1.95; 95%CI,1.48–2.58 times more likely to practice sexual intercourse than students in the age group 17–19 years ,respectively. Kissing, masturbation, and sexual intercourse without condom were higher in those older than 25 years old but age had no statistically significant effect on the practice of anal sex and oral sex. Although gender had no effect on kissing and anal sex, males were found to be more likely to practice sexual intercourse (OR= 2; 95%CI, 1.5–2.67), masturbation (OR= 6.12; 95%CI, 4.23–8.81), oral sex (OR= 1.62; 95%CI, 1.02–2.57) and sexual intercourse without condom (OR= 1.65; 95%CI, 1.16–2.35).

As compared to students of rural origin, students of urban origin were 39% (more likely to practice kissing but 44% less likely to practice sex without condom. On the other hand, the two groups appear to be similar in other sexual behaviors considered in this study.

Respondents who reside outside the campus were more likely to practice sexual intercourse (OR=3.55; 95%CI, 2.42–5.2), kissing (OR=2.86; 95%CI, 1.93–4.22), oral sex (OR=1.85; 95%CI, 1.09–3.14), and sex without condom (OR=2.92; 95%CI, 1.95–4.37) than those who reside in the campus. Yet again, Place of residence had no effect on masturbation and anal sex practices. As Compared to year-one students, year-three and above students were more likely to practice sexual intercourse, kissing, masturbation, and sex without condom. Further more, no difference was observed in the practice of anal sex with year level, but oral sex was found to be 2.14; 95%CI, 1.23–3.66 times more likely among year-one than year-four or above students.

Compared to students of medical sciences, the odds of practicing anal sex and oral sex was higher among students of technology faculty (OR=7.5; 95%CI, 2.96–18.99 and OR=6.23; 95%CI, 3.32–11.67, respectively), and sexual intercourse as well as sex without condom were higher among students of law faculty (OR= 2.53; 95%CI, 1.78–3.6, and OR= 2.9; 95%CI, 1.93–4.35, respectively).

Protestants were less likely to practice sexual intercourse, masturbation, and oral sex than Orthodox Christians by a factor of 0.76 (95%CI, 0.59-.97), 0.74 (95%CI, 0.59–0.94), and 0.59 (95%CI, 0.39–0.90), respectively, and Muslims were less likely to practice masturbation by a factor of 0.71 (95%CI, 0.54–0.92). Religion appears to have no effect on the practice of anal sex or kissing; also Muslims and Christians seem not to be different in practicing sex without condom. Yet, students who had not the habit of attending church/ mosque appear to be more likely to practice sex, kissing, masturbation, and sex without condom by a factor of 2.76 (95%CI, 2.1–3.63), 3.24 (95%CI, 2.44–4.31), 1.94 (95%CI, 1.47–2.57), and 2.32 (95%CI, 1.7–3.16), respectively. On the other hand, religion and church/ mosque attending habit had no effect on anal sex practices.

Sexual intercourse, kissing, and sex without condom were more likely to be practiced among those who were married or had a boy/ girl friend as compared to singles. Oral sex was more likely to be practiced among those who had boy/ girl friend (OR= 1.81, 95%CI, 1.17–2.8) and anal sex was more likely to be practiced among those who were married (OR=4.06, 95%CI, 1.53–10.79) compared to those who were single. Although ethnicity seems to have no effect on the practice of anal sex and oral sex, kissing appears to be more likely practiced among Oromos and Wolytas by a factor of 1.54 (95%CI, 1.25–1.91) and 2.21 (95%CI, 1.25–3.92), respectively as compared to Amharas, still more, Oromos seem less likely to practice masturbation by a factor of 0.76 (95%CI, 0.61– 0.94) as compared to Amharas (table 3).

**Table 3:** Effect of background characteristics of the respondents on their sexual behaviors, Jimma University, August 2009.

Characteristics	Sexual Intercourse OR(95%CI)	Kissing OR (95% CI)	Masturbation OR (95%CI)	Oral Sex OR ( 95%CI)	Anal Sex OR (95%CI)	Sex without condom OR (95%CI)
**Age in years**						
17– 19	1.00	1.00	1.00	1.00	1.00	1.00
20– 24	1.95 (1.48–2.58)^§^	1.27 (1.01– 1.59)*	1.47 (0.87–2.48)	0.86 (0.59– 1.24)	0.85 (0.5– 1.45)	2.21 (1.53–3.18)^§^
25– 45	13.29 (7.67– 23)^§^	4.33 (2.59–7.24)^§^	2.03 (1.58– 2.62)^§^	1.25 (0.6– 2.6)	1.05 (0.35– 3.16)	8.9 (5.11–11.48)^§^
**Gender**						
Male	2 (1.5– 2.67)^§^	1.04 (0.82– 1.31)	6.12 (4.23– 8.81) ^§^	1.62 (1.02– 2.57)*	1.61 (0.82– 3.16)	1.65 (1.16– 2.35) ^Ψ^
Female	1.00	1.00	1.00	1.00	1.00	1.00
**Religion**						
Orthodox	1.00	1.00	1.00	1.00	1.00	1.00
Muslim	1.03 (0.79– 1.35)	1.49 (0.96– 2.32)	0.71 (0.54–0.92)*	0.83 (0.54– 1.26)	1.14 (1.64– 2.01)	1.06 (0.77– 1.47)
Protestant	0.76 (0.59–0.97)*	0.9 (0.7–1.15)	0.74 (0.59–0.94)^Ψ^	0.59 (0.39– 0.9)*	0.79 (0.45– 1.39)	0.79 (0.58– 1.07)
Others^a^	1.55 (0.98– 2.45)	0.92 (0.74– 1.14)	1.01 (0.63–1.63)	0.66 (0.28– 1.54)	0.53 (0.13– 2.2)	1.71 (1.02– 2.87)*
**Place of origin**						
Urban	0.97 (0.79– 1.18)	1.39 (1.16–1.66) ^§^	0.98 (0.81–1.18)	0.94 (0.69–1.28)	1.16 (0.74– 1.81)	1.00
Rural	1.00	1.00	1.00	1.00	1.00	1.44 (1.13–1.84) ^§^
**Level of education**						
Year–one	1.00	1.00	1.00	2.14 (1.23–3.66)^Ψ^	1.00	1.00
Year–two	0.9 (0.67– 1.21)	0.98 (0.76–1.28)	1.24 (0.94–1.63)	1.72 (0.91–3.24)	1.02 (0.57–1.85)	0.89 (0.62– 1.28)
Year–three	1.46 (1.11–1.92^§^	1.78 (1.38–2.31) ^§^	2.38 (1.83– 3) ^§^	0.63 (0.29– 1.39)	0.86 (0.45–1.63)	1.53 (1.11–2.11)*
Year–four/ above	1.83 (1.39–2.41) ^§^	2.5 (1.91–3.26) ^§^	2.1 (1.57–2.73) ^§^	1.00	0.44 (0.19–1.05)	1.68 (1.21–2.33)^Ψ^
**Faculty**						
Medical	1.00	100	1.00	1.00	1.00	1.00
Public health	1.09 (0.81–1.46)	0.9 (0.7– 1.16)	1.18 (0.91– 1.53)	0.87 (0.46–1.66)	0.91 (0.32– 2.57)	1.43 (1.02–2.02)*
Business & economics	1.32 (0.88–1.97)	0.79 (0.55–1.14)	0.5 (0.32– 0.79)^Ψ^	5.47 (3.09–9.67) ^§^	6.3 (2.64– 15.05) ^§^	0.95 (0.55–1.63)
Technology	0.75 (0.43–1.29)	0.68 (0.43– 1.06)	0.95 (0.59– 1.52)	6.23 (3.32–11.67) ^§^	7.5 (2.96– 18.99) ^§^	0.78 (0.39– 1.57)
Humanities &	1.61 (1.22–2.12)^Ψ^	1.21 (0.94–1.56)	1.14 (0.87– 1.49)	3.15 (1.92– 5.18) ^§^	4.69 (2.19–10.05) ^§^	1.13 (0.79– 1.62)
social sciences Law	2.53 (1.78–3.6) ^§^	1.35 (0.97– 1.88)	1.42 (1– 2.02)	2.96 (1.61– 5.42) ^§^	3.02 (1.15– 7.94)*	2.9 (1.93– 4.35) ^§^
Education	2.2 (1.43–3.37) ^§^	0.72 (0.47–1.11)	0.88 (0.56– 1.37)	3.91 (2.01– 7.61) ^§^	5.85 (2.26– 15.1) ^§^	3.05 (1.92–4.95) ^§^
**Place of residence**						
In the university	1.00	1.00	1.00	1.00	1.00	1.00
Out of campus	3.55 (2.42–5.2) ^§^	2.86 (1.93–4.22) ^§^	1.13 (0.76– 1.68)	1.85 (1.09– 3.14)*	1.94 (0.95– 3.98)	2.92 (1.95–4.37) ^§^
**Church/ mosque** **attending habit**						
Yes	1.00	1.00	1.00	1.00	1.00	1.00
No	2.76 (2.1– 3.63) ^§^	3.24(2.44 –4.31) ^§^	1.94 (1.47–2.57) ^§^	1.23 (0.79– 1.91)	1.62 (0.91 (2.88)	2.32 (1.7– 3.16) ^§^
**Marital status**						
Single	1.00	1.00	1.00	1.00	1.00	1.00
Ever married	23.84 (8.4 –7.9) ^§^	10.36 (4 –26.84) ^§^	0.59 (0.28–1.27)	2.35 (0.96– 5.76)	4.06 (1.53– 10.79)^Ψ^	17 (7.55–38.3) ^§^
Have boy/girl friend	3.54 (2.63–4.77) ^§^	7.45 (5.2–10.75) ^§^	1.12 (0.82– 1.52)	1.81 (1.17– 2.8) ^Ψ^	1.46 (0.76–2.81)	3.48 (2.52–4.8) ^§^
**Ethnicity**						
Amhara	1.00	1.00	1.00	1.00	1.00	1.00
Oromo	1.3 (1.03– 1.64)*	1.54 (1.25–1.91) ^§^	0.76 (0.61–0.94)*	0.88 (0.62– 1.25)	1.16 (0.68– 1.95)	1.38 (1.04–1.83)*
Tigre	1.51 (0.85– 2.67)	1.43 (0.83–2.45)	0.68 (0.37–1.25)	1.04 (0.43– 2.52)	1.31 (0.38– 4.49)	1.23 (.6– 2.55)
Gurage	0.94 (0.61– 1.44)	1.24 (0.85–1.82)	0.72 (0.48– 1.09)	0.82 (0.42– 1.61)	1.18 (0.47– 2.95)	0.59 (0.32– 1.09)
Wolayta	1.79 (0.99– 3.22)	2.21 (1.25–3.92)^Ψ^	1.13 (0.63– 2.01)	1.18 (0.48– 2.87)	0.48 (0.06– 3.62)	1.5 (0.74– 3.05)
Others^b^	0.99 (0.71– 1.39)	1.06 (0.78– 1.44)	0.8 (0.58– 1.09)	0.63 (0.35– 1.11)	0.94 (0.43– 2.05)	0.91 (0.59– 1.39)

Othres^a^ include catholic, “waqofetta”, no religion

Others^b^ include Sidama, Keffa, Silte, Agew, Gamo, Somale, Shinasha, Yem, Dawro, Hamer, hadya

Sexual intercourse refers to penis-to-vagina sex

*= p<0 .05, ^Ψ^ =p<0.01, ^§^ =p<0.001

## Discussion

The purpose of this study was to examine the range of sexual practices that exist among university students, their development pattern and whether the development pattern exposes the students to unwanted pregnancy and sexually transmitted infections. Cause-effect relationships could not be established due to the cross sectional nature of the study design. Factors other than socio-demographic characteristics that are known to affect sexual behavior in young people such as pornography were not controlled, and homosexuality which has been known to be a disadvantage for long, was not assessed.

However, the data generated from the large random sample has revealed the range of sexual behaviors that had not been focused by reproductive health planners, health care providers, and researchers in Ethiopia. The analysis of development pattern of the different sexual practices has shown how far the study population is exposed to unwanted pregnancy and sexually transmitted infections, and furthermore, the variation observed in defining ‘sexual intercourse’ has unveiled the need to consciously define the term in its future use in Ethiopia.

Although. more than four-fifth of the study subjects were males and 74.1% were in the age range of 20–24 years, the females participation considered sufficient as it is the reflection of the age and gender composition of the university students. The prevalence of sexual intercourse was consistent with findings of a previous study conducted in Jimma University (15). Other types of sexual practices (kissing, masturbation, oral sex and anal sex) had not been well documented in the Ethiopian context so far. Hggins et al. reported similar practices among college students in the USA, but in a higher proportion (18). The prevalence of these practices in the USA was even much higher among the general public. For example, according to a national estimate among the 25– 44 years, 90% of males and 88% of females had oral sex with an opposite sexual partner and 40% of males and 35% of females had anal sex with opposite sexual partners (19). In our study, it was not possible to differentiate whether anal sex and oral sex were homosexual or heterosexual. Under reporting of these behaviors might have also been the result of strong disapproval towards the act. Anal sex is known to be hidden, stigmatized, and risky with lower rates of reported condom use compared to vaginal sex- even among heterosexuals (20).

The association of sexual practices with seniority in the university, higher age groups, and male gender was observed here and was reported previously (16, 21). In this study, the effect of gender on kissing practice and anal sex as well as the effect of age on anal and oral sex practice was not observed. This could be a result of a balancing effect produced for the lower age groups often make relations with their elders characterized by the absence of power to negotiate. However, anal sex practice was not different between senior and freshman students. On the contrary, oral sex was observed to be more likely among first-year students than senior students. We cannot be sure whether first-year respondents might understand oral sex as ‘just talking about sex. Students from technology and law faculties were more likely to practice risky sexual behaviors than students of medical sciences faculty which could be explained by the differences in their field of study.

The prevalence of kissing was higher among students of urban origin and prevalence of sex without condom was higher among students of rural origin, but the actual differences were small. Students who reside outside the university appear to practice risky sexual behaviors more than students who reside in the university. Whether the desire to practice the sexual behaviors motivated them to reside outside the university, or because they lodge out side the campus, they were pressurized to behave that way could not be explained from our data. However, the decision whether to reside out side the university or in the campus was freely made by the students.

Religion and religious affiliation has been known to affect sexual behavior (3,14,16).As compared to Orthodox Christians, Protestants were less likely to practice sexual intercourse, masturbation, and oral sex, and Muslims were less likely to practice masturbation. Frequent church/mosque attendants had lower rates of risky sexual practices. No difference was observed in anal sex practice among the different religious followers and with church/ mosque attending habit. Although the exclusion of these kinds of sexual behaviors in the religious teachings could be speculated for they are considered as taboo, conclusion could not be tempted without further research.

Although studies revealed the association between ethnicity and sexual behavior (3), there was no difference in the practice of anal and oral sex among the different ethnicities. Higher prevalence of kissing among the Oromo and the Wolyta respondents as compared to Amhara respondents was observed which could be related to existence of subcultures that allow kissing among unmarried youths. The higher masturbation practice observed among Amhara respondents as compared to Oromo respondents needs further data for explanation.

The presence of more sexual practices among those who were ever married and those who had a boy/girl friend is self-explanatory. The presence of anal sexual practices among the ever married more likely than their counter parts needs to be explained by a more focused study.

The finding that 47% of those who practiced oral sex and 29% of those who practiced anal sex did not consider their acts as sexual intercourse agrees with findings in California, USA where 70.6% of adolescents did not consider oral sex as sexual intercourse and 16.1% of the adolescents did not consider anal sex as sexual intercourse (22). It appears that the common use of the term ‘sexual intercourse’ among sex educators and researchers in Ethiopia among similar populations could cause ambiguity.

The sexual behaviors development among the respondents showed a clear pattern when the median ages of practice were observed. They generally start with masturbation at the median age of 17 years. Next, at the median age of 18, they start sexual intercourse, oral sex, and anal sex. After doing so without any condom or any other contraceptives for one year, they start to use condom at a median age of 19 years. Still, after another year, they go into marriage at a median age of 20 years. Generally, contraceptives other than condom are started at the time of marriage. Among those who reported sexual intercourse, 60% had ever practiced it without condom, and 46.6 % practiced it without both condom and contraceptives. Sexual debut in Ethiopia generally starts at marriage (2). These findings revealed that university students are high risk groups for sexually transmitted infections and unplanned pregnancy.

To summarize, masturbation, kissing, penis to vagina sex, oral sex, and anal sex are practiced by Jimma university students. Phone sex, lesbianism and homosexual practices are rumored to exist during the focus group discussions. There appears to be two years risky sexual experience before marriage. Sixty percent of those with sexual experience were exposed to STI and 46.6 % were exposed to both unplanned pregnancy and STI. Forty seven per cent of those who practiced oral sex and 29% of those who practiced anal sex did not consider their behavior as sexual intercourse indicating that the term ‘sexual intercourse’ should be consciously defined for future use in Ethiopian context. These are indicators that show college students are high risk groups that need more focused research and concerted health care. Hence, Service providers and researchers should address all types of sexual practices.
